# Is the Association between Park Proximity and Recreational Physical Activity among Mid-Older Aged Adults Moderated by Park Quality and Neighborhood Conditions?

**DOI:** 10.3390/ijerph14020192

**Published:** 2017-02-14

**Authors:** Jelle Van Cauwenberg, Ester Cerin, Anna Timperio, Jo Salmon, Benedicte Deforche, Jenny Veitch

**Affiliations:** 1 Department of Public Health, Faculty of Medicine and Health Sciences, Ghent University, De Pintelaan 185, Ghent 9000, Belgium; benedicte.deforche@ugent.be; 2 Fund for Scientific Research Flanders (FWO), Egmontstraat 5, Brussels 1000, Belgium; 3 Institute for Health and Ageing, Australian Catholic University, Spring Street 215 (Level 6), Melbourne 3000, Victoria, Australia; Ester.Cerin@acu.edu.au; 4 Institute for Physical Activity and Nutrition, School of Exercise & Nutrition Sciences, Deakin University, Burwood Highway 221, Burwood 3125, Victoria, Australia; anna.timperio@deakin.edu.au (A.T.); jo.salmon@deakin.edu.au (J.S.); jenny.veitch@deakin.edu.au (J.V.); 5 Department of Human Biometry and Biomechanics, Faculty of Physical Education and Physical Therapy, Vrije Universiteit Brussel, Pleinlaan 2, Brussels 1050, Belgium

**Keywords:** motor activity, environment design, retirement, social environment, ecological model, walking

## Abstract

Previous studies have reported mixed findings on the relationship between park proximity and recreational physical activity (PA), which could be explained by park quality and the surrounding neighborhood environment. We examined whether park quality and perceptions of the neighborhood physical and social environment moderated associations between park proximity and recreational PA among mid-older aged adults. Cross-sectional self-reported data on park proximity, park quality, neighborhood physical and social environmental factors, recreational walking and other moderate- to vigorous-intensity recreational physical activity (MVPA) were collected among 2700 Australian adults (57–69 years) in 2012. Main effects between park proximity and measures of recreational PA were non-significant. Park proximity was positively related to engagement in recreational walking among participants who reported average and high social trust and cohesion, but not among those reporting low social trust and cohesion. No other moderating effects were observed. Current findings suggest synergistic relationships between park proximity and social trust and cohesion with mid-older aged adults’ recreational walking. More research is needed to unravel the complex relationship between parks, recreational PA and the social context of neighborhoods.

## 1. Introduction

Promoting physical activity (PA) in mid-older aged adults (55–65 years) is critical to prevent chronic diseases and ensure good quality of life and independent living in older age [1]. Mid-older age may be considered a critical time period for the maintenance or development of PA behaviors since transitional life events (e.g., retirement and children leaving the house) may alter daily routines and habits [2] resulting in additional leisure-time and increased opportunities for recreational PA. Temporary increases in PA have been shown to occur immediately after retirement [3,4]; however, at least 30% of mid-older aged adults living in Westernized countries do not meet the recommended 150 min of moderate- to vigorous-intensity physical activity (MVPA) per week [5].

Socio-ecological models posit that recreational PA is determined by the interaction between the individual and his/her physical and social surroundings. Recreational PA takes place in numerous settings including parks and neighborhood streets, which provide low-cost environments in which to be active. Neighborhoods with easily accessible high-quality parks and supportive physical (e.g., well-maintained walking paths) and social (e.g., positive social trust and cohesion) characteristics are hypothesized to stimulate recreational PA [6]. A recent study among 22- to 65-year-old British adults showed those visiting local green spaces once a week had four times higher odds of being sufficiently physically active compared to those never visiting green spaces [7]. Similarly, among an Australian sample of adults (mean age 56 years), each additional park visit per week was associated with 26% greater odds of engaging in high amounts of leisure-time walking and 11% greater odds of engaging in MVPA [8].

However, several reviews have concluded that knowledge about the relationships between park characteristics and recreational PA among adults and older adults is inconclusive [9,10,11,12]. A study on the relationships of neighborhood open spaces (typically parks) among Australian adults (mean age = 42 years) found availability of an attractive open space to be positively associated with engagement in any recreational walking and meeting MVPA-guidelines by means of recreational walking [13]. In a recent study among Australian and US women (mean age = 34 and 50 years, respectively), the objectively-measured number of parks within 1600 m from home was positively related to meeting MVPA-guidelines among Australian, but not US women. Furthermore, in the same study, distance to the closest park and area of parks within 1600 m from home were not related to women’s MVPA [14]. Importantly, none of these studies focused specifically on mid-older aged adults.

One possible explanation for the mixed findings on the relationship between park proximity and recreational PA may be that park proximity might not relate to recreational PA in all population subgroups or all neighborhood environments. For example, in a previous analysis using data from the current study, we found that non-retired mid-older aged adults who reported living near a park were more likely to participate in recreational walking, whereas no such relationship was observed in retired participants [15]. We observed no such moderating effects for gender, education level, functional limitations and area of residence (urban versus rural). In addition to moderation by individual factors such as retirement status, the relationship between park proximity and recreational PA may depend on physical and social environmental factors. For example, mid-older aged adults might live near a park but not visit the park to be physically active because they perceive the park to be of poor quality or the surrounding neighborhood to be unsafe, they do not trust their neighbors, or perceive the route to the park as hazardous to walk. Although interaction effects between environmental factors are a central premise of socio-ecological models [6], only a limited number of studies have examined moderating effects [16]. In a study of Chinese older adults, Cerin et al. [17] found that the positive relationship between park proximity and recreational PA was stronger when the environment surrounding the park was of higher objectively-measured quality (i.e., did not contain stray animals, signs of crime/disorder, and pollution). Among US older adults, Bracy and colleagues reported the objectively-measured presence of parks to be unrelated to recreational walking. Additionally, the association was not moderated by traffic, pedestrian safety or crime safety [18]. Among Portuguese older adults, objectively-measured proximity of the nearest park was significantly positively related to recreational PA among women and this association was not moderated by objectively-measured crime [19].

In summary, previous studies examining relationships between park proximity, quality and neighborhood characteristics with adults’ and older adults’ recreational PA have yielded inconsistent results and few studies have considered moderation effects between environmental factors. Further, studies including measures of the neighborhood social environment and focusing on mid-older aged adults are particularly scarce. Exploring how park characteristics and neighborhood physical and social environmental factors interact to influence PA is needed to better understand the complex ways in which neighborhood environments shape PA. Therefore, the current study aimed to examine whether the relationship between perceived park proximity and recreational walking and other recreational MVPA among Australian mid-older aged urban and rural adults is moderated by perceived park quality or neighborhood physical (i.e., aesthetics, walking infrastructure, and noise) and social environmental factors (i.e., personal safety, social trust/cohesion and descriptive norms). It was hypothesized that perceived park proximity would positively relate to recreational walking and other recreational MVPA and that these relationships would be stronger when perceived park quality was higher and the perceived neighborhood physical and social environment was more favorable.

## 2. Materials and Methods

### 2.1. Participant Recruitment

The Wellbeing, Eating and Exercise for a Long Life (WELL) study, which has been described in detail elsewhere [20], provided data for the current study. Adults aged 55–65 years were recruited in 2010 (Time 1) and followed up in 2012 (Time 2). The current study used cross-sectional data collected at Time 2 as questions about park proximity and quality were only included at that time point. Participants were initially recruited through stratified cluster random sampling. At Time 1, eighty-four suburbs in Victoria (Australia) were randomly selected within urban-rural and low, medium, and high socio-economic strata. Within each suburb, adults (n = 134, 50% women) within the age range were randomly selected from the electoral roll (compulsory in Australia) and invited to participate via mail. A self-administered postal questionnaire with a reply-paid envelope was mailed to participants one week later. In total, 4082 completed questionnaires were returned (38% response rate) and 3368 (83%) agreed to be re-contacted to participate in a follow-up questionnaire. At Time 2, the questionnaire was completed by 2759 participants (82%). Those who reported not being able to perform PA due to health restrictions or who had moved out of the State of Victoria between Time 1 and Time 2 were excluded. The final analytical sample comprised 2700 participants. The Deakin University Human Research Ethics Committee approved the study protocol.

### 2.2. Measures

The questionnaire was based on an ecological framework which included variables in the individual, social and physical environment domains. The questionnaire assessed the following individual covariates: date of birth, gender, highest education level, marital status, retirement status, and years living at current address. Functional limitations were assessed using the physical functioning scale of the validated Short-Form 36-item Health Survey [21]. Participants were asked how their health limited them in ten activities of daily living (e.g., climbing stairs, washing and dressing, etc.). Response options were: a lot (coded 1), a little (coded 1), or not limited (coded 0) and responses were summed to compute ‘number of functional limitations’. Area-level socio-economic status based on suburb of residence was obtained from the Socio-Economic Index For Areas (SEIFA). SEIFA is a combination of four indexes created from social and economic census information; index of (1) relative socio-economic disadvantage, (2) relative socio-economic advantage and disadvantage, (3) economic resources and (4) education and occupation [22]. Suburbs were classified as urban, rural, or fringe consistent with the Australian Regional Infrastructure Development Fund Act 1999 [23,24]. Although participants were not recruited from ‘fringe’ areas at Time 1, at Time 2, 45 participants had moved to a fringe suburb (within Victoria); these participants were retained in the analyses.

Using a question from the Neighborhood Environment Walkability Scale (NEWS) [25], participants indicated how long it would take them to walk from their home to the nearest park using a five-point scale; 1–5 min, 6–10 min, 11–20 min, 21–30 min and + 31 min (and an additional “don’t know”-option). Standard scoring protocols were applied (available on http://www.drjamessallis.sdsu.edu/measures.html) [25]. Satisfaction with park quality was assessed by asking participants how much they (dis)agreed with the following statement: ‘I am satisfied with the quality of parks in my neighborhood’. Response options were a five-point Likert scale ranging from (1) ‘strongly disagree’ to (5) ‘strongly agree’. This variable acted as an indicator of perceived park quality.

Three physical neighborhood environmental factors were examined: aesthetics, walking infrastructure, and noise. These variables were derived from validated questionnaires [25,26] and were created by averaging the scores obtained from a five-point Likert scale ranging from (1) ‘strongly disagree’ to (5) ‘strongly agree’. Items related to aesthetics (six) included: ‘There is a lot of rubbish on the street in my neighborhood’ (reverse coded); ‘In my neighborhood the buildings and homes are well-maintained’; ‘The buildings and homes in my neighborhood are interesting’; ‘My neighborhood is attractive’; ‘The trees in my neighborhood provide enough shade’; and ‘It is pleasant to walk in my neighborhood’. Items related to walking infrastructure (three) included: ‘The footpaths in my neighborhood are in good condition’; ‘The streets in my neighborhood are well lit at night’; and ‘In my neighborhood it is easy to walk to places’. The presence of noise was assessed by a single item: ‘There is a lot of noise in my neighborhood’ (reverse coded).

Three social neighborhood environmental factors were examined: personal safety, social trust/cohesion and descriptive norms. Items related to personal safety (three) included [26]: ‘I feel safe walking in my neighborhood, day or night’; ‘Violence is not a problem in my neighborhood’; and ‘My neighborhood is safe from crime’. Social trust/cohesion was assessed using five items [27]: ‘Most people can be trusted’, ‘This is a close-knit neighborhood’, ‘People around here are willing to help their neighbors’, ‘People in this neighborhood generally don’t get along with each other’ (reverse coded), and ‘People in this neighborhood do not share the same values’ (reverse coded). Descriptive norms was assessed using two items [26]: ‘I often see other people walking in my neighborhood’ and ‘I often see other people exercising in my neighborhood’. All items had a five-point Likert response scale ranging from (1) ‘strongly disagree’ to (5) ‘strongly agree’ with the item-scores being averaged.

Participants completed the International Physical Activity Questionnaire (long version, IPAQ-L) to assess recreational walking and other recreational MVPA. The IPAQ-L has been shown to have excellent test-retest reliability and acceptable validity in adults aged 15–69 years [28]. Participants indicated frequency (number of days) and average duration on one of those days (hours and minutes) they walked for recreation for at least 10 consecutive minutes during the last seven days, which were multiplied to compute total ‘recreational walking’. Participation in other types of recreational MVPA (excluding walking) was assessed and calculated similarly. Both variables were expressed in minutes per week. For descriptive purposes, the variables recreational walking and other recreational MVPA were summed to obtain an overall measure of recreational PA.

### 2.3. Statistical Analyses

All statistical analyses were conducted using Stata 10.1 with statistical significance defined at α = 0.05. To account for clustering of participants within suburbs, regression models with Huber-White sandwich (robust) standard errors were used as described by Cerin [29]. More specifically, zero-inflated negative binomial (ZINB) regression models with robust standard errors were used to analyze the relationships of perceived park proximity, park quality, and neighborhood physical and social environmental factors with recreational walking and other recreational MVPA. ZINB models were used because the outcomes were positively skewed with excessive zero values. The need for zero-inflated regression models was confirmed by Vuong tests [30]. ZINB models evaluate two relationships simultaneously: (1) The relationships between the independent variables and the odds of non-participation in recreational walking/other recreational MVPA (odds ratio, OR) and (2) The relationships with weekly minutes of recreational walking/other recreational PA for participants who did engage in some recreational walking/other recreational MVPA (negative-binomial model regression coefficient representing proportional change).

First, a basic model including the main effects of perceived park proximity, quality, and all neighborhood physical and social environmental factors was fitted. Second, seven separate models were estimated which added the two-way interaction effects of perceived park proximity with perceived park quality, the three neighborhood physical and three neighborhood social environmental factors to the basic model. Third, a final model combining the basic model with all significant interaction effects observed in the previous step was constructed. In case of a significant interaction effect, the relationship between park proximity and recreational physical activity was estimated at the mean value of the moderator and at this mean plus and minus one standard deviation (these were referred to as average, below average and above average levels of the moderator, respectively) [31]. In a previous study using the same dataset, we found park proximity to interact significantly with retirement status [15]. Therefore, when we observed a significant two-way interaction effect in the current study, we also analyzed the three-way interaction effect with retirement status to check whether the results were applicable for retired as well as non-retired mid-older aged adults. Given their relationship with recreational PA in previous research [15], age, gender, education, retirement status and functional limitations were included as covariates in all analyses, as were area-level SES and area of residence. Recreational MVPA was included as a covariate in analyses with recreational walking as the dependent variable (and vice versa).

## 3. Results

### 3.1. Descriptive Statistics

Participants had a mean age of 62.3 (±3.1) years, 52.9% were women, almost a third (29.9%) had obtained a university degree, most (78.1%) lived with a partner, and 44.3% were retired. Median years living at the current address was 15.0 (interquartile range: 7.0–28.1 years) and participants reported a mean of 2.7 (±2.6) functional limitations (out of 10 activities). About half of the participants (52.2%) were living in a rural area. Environmental perceptions were generally favorable (see Table 1). The median value of walking for recreation was 70.0 min/week (interquartile range: 0.0–210.0) and that of non-walking recreational MVPA was 0 min/week (interquartile range: 0.0–120.0). The median value of overall recreational PA was 150 min/week (interquartile range: 0.0–360.0), which implies that half of the participants met the recommended amount of 150 min/week through recreational PA.

### 3.2. Relationships between Park Proximity and Recreational Pa and Moderating Effects of Park Quality and Physical and Social Neighborhood Environmental Factors

The results for the relationships with recreational walking are presented in Table 1. The odds ratios of the logit model represent the likelihood of non-participation in recreational walking. None of the odds ratios for the main effects were statistically significant. However, a significant interaction effect between perceived park proximity and social trust/cohesion was observed (data not shown). For average and above average (1 standard deviation above average) social trust/cohesion, higher perceived park proximity was related to decreased odds of non-participation in recreational walking (OR = 0.93, 95% CI = 0.87, 0.99 and OR = 0.85, 95% CI = 0.78, 0.94, respectively). In other words, for average and above average levels of social trust/cohesion, those who perceived they lived 1 unit closer to a park had respectively 1.08 (1/0.93 = 1.08 with 95% CI = 1.01, 1.15) and 1.18 (1/0.85 = 1.18 with 95% CI = 1.06, 1.28) times higher odds of walking for recreation. For below average social trust and cohesion (1 standard deviation below average), perceived park proximity was not significantly related to participation in recreational walking (OR = 1.01, 95% CI = 0.92, 1.11). The three-way interaction effect park proximity * social trust/cohesion * retirement status was non-significant.

For those who engaged in recreational walking, the parameters in the negative binomial model represent the proportional increase in recreational walking for each one-unit increase in the predictor. Significant positive relationships were observed for perceived park quality and social trust/cohesion. For those who participated in recreational walking, a one-unit increase in perceived park quality was related to a 2% increase in minutes of recreational walking. Likewise, a one-unit increase in social trust/cohesion was related to a 13% increase in minutes of recreational walking. No significant interaction effects were observed in the negative binomial model.

For other types of recreational MVPA, there were no significant main or interaction effects observed in the logit or the negative binomial models.

## 4. Discussion

Among our sample of mid-older aged adults (of which 44% were retired), we found perceived park proximity to be related to participation in recreational walking if participants reported social trust/cohesion to be average or above. This partially confirms our hypothesis stating that relationships of park proximity with recreational PA would be stronger when the neighborhood environment is favorable for PA. Similar synergistic effects were observed among Hong Kong older adults (aged ≥ 65 years) for whom the absence of stray animals, signs of crime/disorder and pollution was found to reinforce the positive relationship between park proximity and recreational PA [17]. From a policy and practice viewpoint these findings imply that the mere provision of parks may be insufficient to stimulate recreational PA. It should be accompanied by strategies fostering upkeep, feelings of safety and social trust and cohesion. However, in the current study as well as in previous studies among US [18] and Portuguese [19] older adults no moderating effects between the presence of parks and measures of safety from crime on recreational PA were reported.

Previous studies examining relationships of social trust/cohesion with walking among adults [32,33] and older adults [34,35,36,37] have yielded inconsistent results. Our findings suggest that high levels of social trust/cohesion might contribute to higher levels of recreational walking through: (1) Moderating the relationship between park proximity and participation in recreational walking; and (2) Its relationship with higher volumes of recreational walking among those who already walk. Mid-older aged adults with higher levels of social trust/cohesion might be stimulated to use nearby parks to walk and may walk more because they experience increased social support to walk and enjoy encounters with people in their neighborhood during their walks [38]. These findings illustrate the importance of examining both physical and social neighborhood environmental factors as well as the interaction effects between park proximity and these neighborhood environmental factors. In a previous study using the same dataset, we found that park proximity interacted significantly with retirement status; non-retired participants who reported living near a park were more likely to participate in recreational walking, whereas no relationship was observed among retired participants [15]. In the current study, there was no evidence of a three-way interaction effect between park proximity, social trust and cohesion and retirement status. Hence, current findings are applicable to retired as well as non-retired participants. It should also be noted that in our previous study using this dataset, the main effect analyses showed perceived park proximity to be significantly related to the odds of participating in recreational walking whereas this relationship was (borderline) non-significant in the current study (*p* = 0.05). This difference may be explained by the additional adjustment for neighborhood physical and social environmental factors in the current study. Based on findings from the previous study, one might conclude that park proximity is not important for retired mid-older aged adults’ recreational walking, whereas the current study shows that park proximity does relate to engagement in recreational walking among retirees but only when social trust/cohesion is high.

The observed main effects showed that higher park quality and social trust/cohesion were related to proportionally higher levels of recreational walking among those who walk for recreation. This is consistent with previous research among Australian [13] and Canadian [39] adults, which showed proximity-measures to be related to engaging in any recreational walking or MVPA whereas quality-measures (attractiveness and size) were related to achieving higher volumes of PA. Hence, having a park nearby might stimulate adults to walk to and within this park (when social trust and cohesion is sufficiently high), but higher park quality might encourage adults who already walk for recreation to spend a longer period of time in the park and accumulate more minutes of walking. In addition, a recent study among Australian adults, showed quality of public open spaces, operationalized as the objective presence of features such as grassed areas, amenities and dog-related facilities, to be related to the odds of recreational walking to a public open space [40].

Our findings highlight the potential importance of social environmental factors which have received considerably less research attention than physical environmental factors [38,41] and should be a stronger focus of future research. We observed no significant relationships between neighborhood physical environmental factors and recreational walking or other recreational MVPA. This supports the notion that physical neighborhood characteristics may be less relevant for recreational walking and MVPA (which can be performed anywhere) as opposed to transport-related PA (which usually originates from the home) [42]. Recreational MVPA other than walking may require specific skills or a certain level of fitness rendering these activities more susceptible to individual (e.g., self-efficacy, perceived competence, fitness level) rather than environmental factors. Furthermore, such activities may require specific facilities (e.g., swimming pool, tennis courts) and the presence of such facilities in the neighborhood or park were not assessed in the current study. Mid-older aged adults that are motivated to engage in MVPA other than walking may also be willing to travel to specific facilities located outside their neighborhood to engage in these activities. Future studies should include individual as well as environmental measures of specific recreational or sports facilities to better understand the correlates of recreational MVPA other than walking.

Strengths of the current study include the large sample size; the focus on recreational PA among mid-older aged adults; the inclusion of urban and rural residents; the purposeful variation in area-level SES; and the examination of the importance of perceived park proximity, park quality, neighborhood physical and social environmental factors and associated interactions. However, the study was limited by its cross-sectional design and, therefore, cause and effect relationships cannot be determined. For example, it might be that higher levels of walking might promote social interactions among neighbors, as was shown previously [43], rather than social environmental factors stimulating walking. Furthermore, we exclusively relied upon subjective measures of park proximity, park quality, and physical and social environmental factors. However, previous research has shown that subjective measures may be more strongly related to PA behaviors than objective measures of the environment. For example, among Danish 18-to-80-year-olds, Schipperijn and colleagues [44] found subjective distance to the nearest park to be more strongly related to park use than objectively-measured distance. However, the same study also showed that the nearest park is not always the most frequently used park. Future research would benefit from including subjective and objective measures of park proximity as well as taking into account other measures of park availability (e.g., number and size of parks or total park area in the neighborhood). Satisfaction with park quality was used as an indicator of perceived park quality which was not assessed directly. Future studies should include more detailed measures of perceived and objective park quality, and audits of actual park facilities, amenities and programs as well as the presence/proximity of other neighborhood destinations. Lastly, we examined overall recreational walking and other recreational MVPA without specifying the context where the activities occurred. Future studies could use context-specific measures of recreational PA (e.g., park-based recreational walking) or combine accelerometry with GPS-devices to more specifically match the environmental exposures with the PA outcomes.

## 5. Conclusions

Our findings suggest that fostering social trust and cohesion among neighbors may stimulate mid-older aged adults to use nearby parks for recreational walking. Additionally, fostering social trust and cohesion and the provision of high-quality parks may promote more walking among mid-older aged adults who already walk for recreation. The presence of specific facilities other than parks (i.e., gyms, sports fields or clubs) may be necessary to promote other recreational physical activities (e.g., jogging, cycling and sports activities) among mid-older aged adults. More research is needed to unravel the complex relationships between park usage, neighborhood physical and social environmental characteristics and recreational PA among mid-older aged adults.

## Figures and Tables

**Table 1:** Descriptive statistics and main effects of perceived park proximity and the potential moderators on recreational walking and other recreational MVPA.

	Descriptives	Relationships with Recreational Walking ^a^		Relationships with Other Recreational MVPA ^b^	
	Mean ± SD (on a 5-Point Scale)	Logit Model ^c^: OR of Being a Non-Walker (95% CI)	Negative Binomial Model ^d^ (95% CI)	Logit Model ^c^: OR of No Other Recreational MVPA (95% CI)	Negative Binomial Model ^d^ (95% CI)
Perceived park proximity	3.9 ± 1.3	0.93 (0.87, 1.00)	1.01 (0.97, 1.06)	0.99 (0.92, 1.06)	0.97 (0.92, 1.03)
Perceived park quality	3.8 ± 0.9	1.01 (0.98, 1.04)	1.02 (1.01, 1.02) **	0.91 (0.83, 1.00)	0.99 (0.90, 1.09)
Physical neighborhood environment					
Aesthetics	3.9 ± 0.5	0.86 (0.66, 1.11)	0.93 (0.82, 1.06)	1.11 (0.88, 1.39)	1.05 (0.89, 1.23)
Walking infrastructure	3.5 ± 0.8	1.03 (0.88, 1.20)	1.00 (0.93, 1.08)	1.07 (0.93, 1.24)	1.05 (0.94, 1.18)
Noise	3.8 ± 1.0	1.06 (0.95, 1.18)	0.97 (0.92, 1.03)	1.00 (0.89, 1.12)	0.96 (0.90, 1.03)
Social neighborhood environment					
Personal safety	3.7 ± 0.8	1.01 (0.88, 1.15)	1.02 (0.95, 1.09)	1.03 (0.90, 1.18)	1.03 (0.94, 1.13)
Social trust and cohesion	3.6 ± 0.5	0.90 (0.74, 1.08)	1.13 (1.01, 1.26) *	0.97 (0.77, 1.22)	0.94 (0.82, 1.06)
Descriptive norms	4.1 ± 0.7	0.86 (0.74, 1.01)	1.10 (0.99, 1.22)	0.87 (0.75, 1.00)	1.00 (0.92, 1.10)
Neighborhood-level covariates					
Area of residence					
Urban (=ref., %)	46.1	/	/	/	/
Rural (%)	52.2	0.87 (0.66, 1.13)	1.03 (0.92, 1.15)	1.09 (0.85, 1.93)	0.92 (0.78, 1.09)
Fringe (%)	1.7	0.87 (0.41, 1.86)	0.84 (0.64, 1.12)	1.33 (0.69, 2.53)	0.77 (0.50, 1.19)
Socio-economic status ^e^	988.1 ± 67.5	1.00 (1.00, 1.00)	1.00 (1.00, 1.00)	1.00 ^f^ (1.00, 1.00) *	1.00 (1.00, 1.00)

SD = standard deviation; CI = confidence interval; OR = odds ratio; * *p* < 0.05; ** *p* < 0.001; ^a^ The model for recreational walking included 2291 observations, of which 755 were zero observations, and was adjusted for age, gender, education, retirement status, functional limitations, suburb SES, area of residence, and other recreational MVPA. Other recreational MVPA was significantly related to the odds of being a non-walker (OR = 0.998, 95% CI = 0.998, 0.999, *p* < 0.001) and the amount of recreational walking (ExpB = 1.0004, 95% CI = 1.0002, 1.0005, *p* < 0.001) among those who walked for recreation; ^b^ The model for other recreational MVPA included 2291 observations, of which 1404 were zero observations, and was adjusted for age, gender, education, retirement status, functional limitations, suburb SES, area of residence, and recreational walking. Recreational walking was significantly related to the odds of no other recreational MVPA (OR = 0.999, 95% CI = 0.999, 1.000, *p* = 0.023) and the amount of other recreational MVPA (ExpB = 1.0008, 95% CI = 1.0005, 1.0010, *p* < 0.001) among those who engaged in other recreational MVPA; ^c^ In the logit model, the relationships between the independent variables and the odds of non-participation in recreational walking or other recreational MVPA were estimated; ^d^ In the negative binomial model, the relationships with weekly minutes of recreational walking or other recreational MVPA for participants who did engage in some recreational walking or other recreational MVPA were estimated. Negative binomial model parameters represent the proportional increase in minutes/week recreational walking or other recreational MVPA with a one-unit increase in the predictor; ^e^ Higher scores represent higher levels of neighborhood socio-economic status; ^f^ Exact odds ratio was 0.998, implying that a one-unit higher score on socio-economic status was associated with a 0.2% lower odds of no other recreational MVPA.
